# Expanding the Genotype-Phenotype Correlations and Mutational Spectrum in Inherited Retinal Diseases: Novel and Recurrent Mutations

**DOI:** 10.7759/cureus.53742

**Published:** 2024-02-06

**Authors:** Ibrahim Sahin, Haktan B Erdem, Taha Bahsi, Hanife Saat

**Affiliations:** 1 Department of Molecular Medicine, College of Medicine and Medical Sciences, Arabian Gulf University, Manama, BHR; 2 Department of Medical Genetics, University of Health Sciences, Dışkapı Yıldırım Beyazıt Training and Research Hospital, Ankara, TUR; 3 Department of Medical Genetics, Ankara Etlik City Hospital, Ankara, TUR

**Keywords:** ngs, leber congenital amaurosis, usher syndrome, stargardt syndrome, retinopathy, retinitis pigmentosa, retina

## Abstract

Background

Inherited retinal diseases (IRD) represent a prominent etiology of visual impairment on a global scale. The lack of a clear definition of the etiology and genotypic spectrum of IRD is attributed to the significant genetic variability seen. Additionally, there is a scarcity of available data about the correlations between genotypes and phenotypes in this context. This study aimed to clarify the range of mutations and the associations between genotypes and phenotypes in IRD.

Methods

This cohort consists of 223 patients who have been diagnosed with a range of retinal illnesses, such as retinitis pigmentosa (RP), Stargardt (STGD)/STGD-like disease, Usher syndrome, and Leber congenital amaurosis (LCA). The validation of each mutation and its pathogenicity was conducted by bioinformatics analysis, Sanger sequencing-based co-segregation testing, and computational assessment. The link between genotype and phenotype was analyzed in all patients who possessed mutations as described in the recommendations established by the American College of Medical Genetics.

Results

A total of 223 cases, comprising Turkish and Syrian families, were examined, revealing the presence of 175 distinct mutations in the IRD gene. Among these mutations, 58 were identified as unique, indicating that they had not been previously reported. A total of 119 mutations were identified to be likely pathogenic, while 104 mutations were classified as pathogenic. The study identified patterns of heredity, namely autosomal recessive, dominant, and X-linked inheritance.

Conclusions

The findings of this study broaden the clinical and molecular aspects of IRD and further enhance our understanding of its complex nature. The discovery of previously unknown relationships between genetic variations and observable traits, as well as the wide range of genetic variants associated with IRD, significantly contributes to our existing understanding of the diverse phenotypic and genotypic characteristics of IRD. This new information will prove invaluable in facilitating accurate clinical diagnoses as well as personalized therapeutic interventions for individuals affected by IRD.

## Introduction

Inherited retinal diseases (IRD) encompass a group of monogenic conditions that result in impaired vision due to retina degeneration. IRD encompass retinitis pigmentosa (RP) and related disorders. The prevalent manifestation involves a pathological alteration in the photoreceptor cells, specifically the rod and/or cone cells, which are specialized neurons in the retina that are sensitive to light [1,2].

IRD exhibit an approximate global frequency ranging from 1 in 2000 to 3000 persons. RP is common, with a prevalence rate of around 1 in 3500 persons [3,4]. RP is a hereditary retinal disorder characterized by a gradual degeneration of rod and cone photoreceptor cells, resulting in a sequential loss of visual functions. Typically, individuals with RP have a decline in night vision during adolescence, followed by a reduction in peripheral vision in early adulthood, and ultimately, a deterioration of central vision in later life. The electroretinogram and other retinal function measures indicate a general decline in photoreceptor function several years prior to the onset of symptomatic conditions such as night blindness, visual-field scotomas, or impaired visual acuity [2].

At present, RP lacks a standard classification that is universally recognized. The classification of RP is based on many key variables. These elements include the topography of the diseased retina, which can be categorized as central, pericentral, sector, or peripheral. Another criterion is the age at which the ailment first manifests. Additionally, the method of inheritance is considered in the classification process. The main type of photoreceptors implicated in the disease is also taken into account. A systemic variant of RP manifests in several tissues and organs, whereas nonsyndromic or simple variants primarily impact the eye alone. Syndromic variants of a condition have been seen to impact supplementary physiological systems, including the auditory system. The prevailing consensus in the field acknowledges that around 70-80% of all instances of RP may be categorized as nonsyndromic rod-cone dystrophy [4].

RP comprises a variety of illnesses that exhibit unique etiologies and separate molecular mechanisms yet have common symptoms and effects. In addition to uncommon mitochondrial and digenic variants, the inheritance patterns of this condition include autosomal-dominant (accounting for around 30-40% of cases), autosomal-recessive (50-60%), and X-linked (XL) (5-15%) types [2]. RP can manifest either in isolation or as a component of a more intricate disease. The nature of basic RP exhibits a notable level of complexity. Every genetic variant is the result of mutations occurring in many genes, often numerous. Numerous distinct mutations exhibiting comparable outcomes have been identified for the majority of genes; nonetheless, it is noteworthy that certain mutations within the same gene can give rise to disparate disorders. It is noteworthy that a particular mutation can elicit divergent symptoms among people, including those within the same familial context [5].

Usher’s syndrome, characterized by the co-occurrence of RP and hearing impairment, is the most prevalent syndromic variant, including around 20-40% of persons affected by the recessive form of the illness (or 10-20% of the total cases). Bardet-Biedl syndrome is a significant variant of syndromic RP, whereby RP is observed in conjunction with varying degrees of obesity, cognitive impairment, polydactyly, hypogenitalism, and renal illness [2].

Approximately 300 disease-causing genes linked to IRD have been discovered, as reported by RetNet (https://web.sph.uth.edu/RetNet/home.htm). This extensive genetic heterogeneity poses significant challenges for molecular diagnosis, as it requires considerable time and resources to accurately identify the underlying genetic mutations [6]. Over 200 causal genes have been documented, with a total of over 4500 mutations reported (RetinoGenetics: http://www.retinogenetics.org/) [7].

Gene therapy is a feasible treatment option for retinal abnormalities, primarily because the eye is well-suited for direct intraocular injection. Moreover, the eye has immunological privilege, a characteristic that allows it to tolerate the presence of foreign antigens without eliciting an immune or inflammatory reaction. This particular attribute has the potential to reduce the likelihood of negative inflammatory responses and/or rejection by vectors. In addition, it is possible to treat each eye separately, allowing for the assessment of efficacy in one eye before proceeding with the other, thus reducing the risk of visual impairment. In December 2017, the US Food and Drug Administration (FDA) provided approval for voretigene neparvovec-rzyl (Luxturna™), a gene therapy developed explicitly for the treatment of vision loss and Leber congenital amaurosis 2 (LCA2), a congenital retinal degenerative disorder. This approval is intended for patients who have been diagnosed with confirmed biallelic pathogenic variants in the *RPE65* gene [8]. In the course of clinical investigations, individuals who received this substance and were monitored for a duration of up to nine years reported improvements in their visual capabilities, while not experiencing any notable negative consequences related to the treatment. A novel gene therapy construct has been created to specifically target people suffering from XL RP caused by biallelic pathogenic mutations in the *RPGR* gene [8,9].

Next-generation sequencing (NGS) has become a powerful and important approach for simultaneously investigating many genes. Its widespread adoption in molecular diagnostics has been rapid [10].

This study involved a cohort of 223 individuals who underwent NGS in order to assess the importance of the IRD phenotype and clinical variability. The results of our study broaden the existing range of gene mutations and offer novel perspectives on the relationship between genotypes and phenotypes in IRD.

## Materials and methods

Patients

Informed consent has been obtained from the patients or their parents to publish the research findings and any further linked materials. Data were collected from a total of 223 patients who were referred to our clinic. During the period spanning from 2017 to 2021, individuals were subjected to either the Sophia Hereditary Disease Solution (HDS) panel or clinical exome sequencing (CES) at the Ankara Central Genetic Laboratory in Turkey.

DNA extraction

The genomic DNA of the patients was isolated from blood samples collected in ethylenediaminetetraacetic acid (EDTA) tubes using the QIAamp DNA Blood Midi Kit (Qiagen Inc., Hilden, Germany) in accordance with the manufacturer's guidelines. For DNA sample quantification, a Qubit 4 fluorometer and NanoDrop 1000 spectrophotometer (both manufactured by Thermo Fisher Scientific Inc., MA, USA) were utilized.

NGS

The IRD panel was carried out utilizing the Sophia HDS Kit (Sophia Genetics, Saint-Sulpice, CH), while the CES panel was executed on the Illumina NextSeq 500 (Illumina Inc., San Diego, USA) using the Sophia Clinical Exome Kit (Sophia Genetics, Saint-Sulpice, CH).

NGS interpretations

Utilizing SOPHiA-DDM-v4 software (Sophia Genetics, Saint-Sulpice, CH), the data were analyzed. The NGS methodology has been assessed and fine-tuned on the Sophia-DDM-v4 platform in order to achieve enough sequencing depth for the identification of deletions and duplications.

The variations underwent filtration using both the Genome Aggregation Database and our own developed database. The VarSome and Franklin tools (Genoox, Tel Aviv, IL) were utilized to apply several pathogenic prediction methods to variations. The analysis of splicing variants was conducted using the Human Splicing Finder. A comprehensive search was conducted on the ClinVar and Leiden Open Variation Databases to identify documented instances of potentially pathogenic genetic variants associated with diseases. The disease-causing mutations were confirmed by the utilization of Sanger sequencing, and subsequent analysis was conducted to determine their segregation. The primers utilized in this study were developed and manufactured by Oligomer Biotechnology, a reputable company specializing in oligonucleotide synthesis (https://oligomer.com.tr). According to the guidelines set out by the American College of Medical Genetics and Genomics, variations were categorized into several classes, including pathogenic, likely pathogenic, variant of unknown significance (VUS), likely benign, and benign [11]. This study includes variants that were classified as pathogenic or likely pathogenic. The data were visualized with IGV 2.16.2 software (Broad Institute, Cambridge, USA).

Descriptive statistics and graphics

Descriptive statistical analyses were computed, and visual representations were generated using Python 3.11.6.

## Results

**Figure 1:**
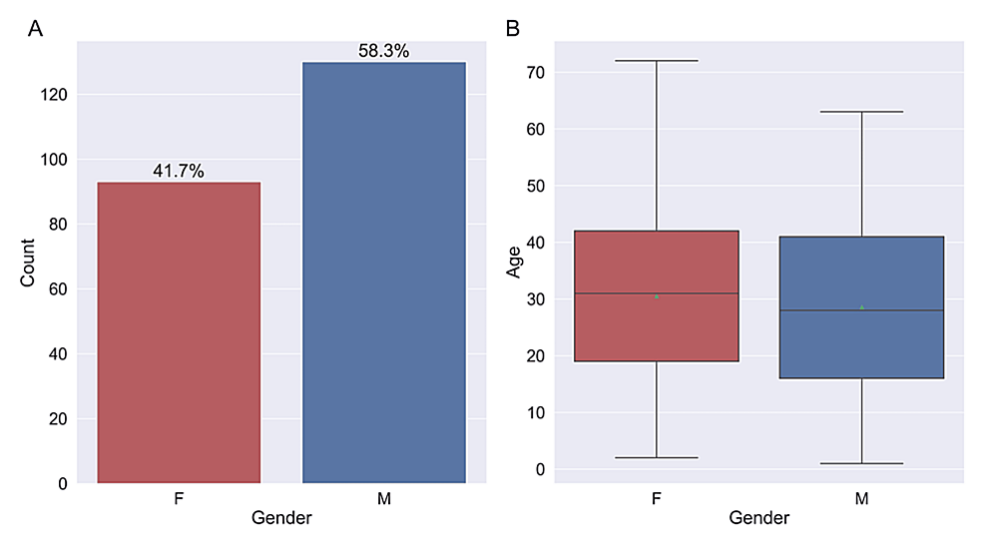
Patients’ characteristics (A) Bar plot showing the number and percentage of patients in terms of gender. (B) Boxplot showing the mean (green triangle) and median (black line) age of the patients in terms of gender. M: Male, F: Female

The majority of patients (194, 87%) presented at our clinic with a prediagnosis of RP. The mean age of the participants was 29.4 years, with a range spanning from 1 to 72 years. The male participants (n = 130, 58.3%) outnumbered the female participants (n = 93, 41.7%) by a significant margin (Figure 1).

**Figure 2:**
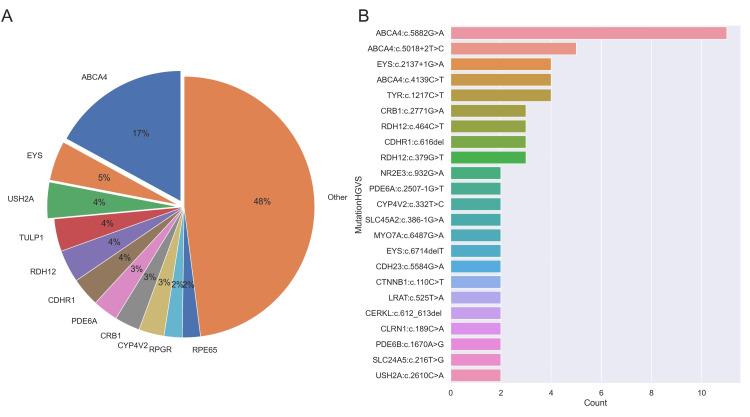
Spectrum of the genes and mutation types (A) Pie chart showing the spectrum of the mutated genes (top 11 genes and others) with percentages in the study. (B) Bar plot showing the mutation types in the study.

A total of 175 distinct mutations associated with IRD were identified in the observed cases. Out of the total sample size, 151 individuals exhibited homozygosity, accounting for 67.7% of the population. Heterozygosity was observed in 64 individuals, representing 28.7% of the sample. Hemizygosity was identified in six individuals, comprising 2.7% of the population. Lastly, compound heterozygosity was observed in two individuals, making up 0.9% of the sample. These findings are summarized in Table 1 and visually shown in Figure 2.

**Table 1:** Demographic features and mutations of the patients MutationHGVS: variant naming according to Human Genome Variation Society, ACMG: The American College of Medical Genetics and Genomics (ACMG)-recommended variant classification, Mut: variant defined according to the coding region, F: female, M: male, RP: retinitis pigmentosa, P: pathogenic, LP: likely pathogenic, AR: autosomal recessive, AD: autosomal dominant, XL: X-linked

Patient	Gender	Indication	Age	MutationHGVS	Zygosity	ACMG	Novelty	Inheritance Pattern	Associated Conditions	Gene	Mut
1	F	RP	9	NR2E3:c.932G>A	heterozygous	LP	no	AR/AD	Vitreoretinal Degeneration, Retinitis Pigmentosa	NR2E3	c.932G>A
2	F	RP	35	PROM1:c.1052del	heterozygous	LP	yes	AR/AD	RP	PROM1	c.1052del
3	M	RP	41	EYS:c.2137+1G>A	homozygous	P	no	AR	RP	EYS	c.2137+1G>A
4	F	RP	47	CRB1:c.2771G>A	heterozygous	P	no	AR/AD	RP	CRB1	c.2771G>A
5	F	RP	51	EYS:c.2137+1G>A	homozygous	P	no	AR/AD	RP	EYS	c.2137+1G>A
6	M	RP	42	ABCA4:c.4139C>T	homozygous	P	no	AR/AD	RP	ABCA4	c.4139C>T
7	M	RP	5	TULP1:c.1318C>T	homozygous	P	no	AR	RP	TULP1	c.1318C>T
8	F	RP	2	MYO7A:c.1117C>T	homozygous	LP	no	AR	Usher Syndrome	MYO7A	c.1117C>T
9	M	RP	38	CERKL:c.235_238del	homozygous	LP	yes	AR	RP	CERKL	c.235_238del
10	M	RP	43	RDH12:c.481C>T	homozygous	LP	no	AR	RP	RDH12	c.481C>T
11	M	RP	24	RPE65:c.138del	homozygous	P	no	AR/AD	RP	RPE65	c.138del
12	M	RP	35	ABCA4:c.4234C>T	heterozygous	P	no	AR/AD	RP	ABCA4	c.4234C>T
13	M	RP	50	ABCA4:c.2014A>G	heterozygous	LP	no	AR/AD	RP	ABCA4	c.2014A>G
14	M	RP	14	USH2A:c.11864G>A	homozygous	P	no	AR/AD	Usher Syndrome	USH2A	c.11864G>A
15	F	RP	44	ABCA4:c.5882G>A	heterozygous	LP	no	AR/AD	RP	ABCA4	c.5882G>A
16	M	RP	29	ABCA4:c.4225A>G	homozygous	LP	no	AR/AD	RP	ABCA4	c.4225A>G
17	F	RP	13	CEP290:c.5668G>T	heterozygous	P	no	AR/AD	RP	CEP290	c.5668G>T
18	F	RP	47	RPE65:c.917C>T	homozygous	P	no	AR/AD	RP	RPE65	c.917C>T
19	M	RP	44	ABCA4:c.4139C>T	homozygous	P	no	AR/AD	RP	ABCA4	c.4139C>T
20	F	Stargardt	28	ABCA4:c.2254dupA	homozygous	LP	yes	AR/AD	RP	ABCA4	c.2254dupA
21	F	RP	35	CDHR1:c.616del	homozygous	P	yes	AR	RP	CDHR1	c.616del
22	F	RP	41	CYP4V2:c.253C>T	homozygous	LP	no	AR	Bietti Crystalline Corneoretinal Dystrophy	CYP4V2	c.253C>T
23	F	RP	19	ADGRV1:c.5361C>A	homozygous	LP	yes	AR	Usher Syndrome	ADGRV1	c.5361C>A
24	F	RP	56	ABCA4:c.5714+1G>T	homozygous	P	no	AR/AD	RP	ABCA4	c.5714+1G>T
25	F	RP	37	RPE65:c.271C>T	homozygous	P	no	AR/AD	RP	RPE65	c.271C>T
26	M	RP	39	MERTK:c.2190-1G>T	homozygous	P	yes	AR/AD	RP	MERTK	c.2190-1G>T
27	M	RP	36	ABCA4:c.3608G>A	heterozygous	LP	no	AR/AD	RP	ABCA4	c.3608G>A
28	M	RP	27	EYS:c.490C>T	homozygous	P	no	AR/AD	RP	EYS	c.490C>T
29	M	RP	30	RP2:c.264_271del	hemizygous	LP	yes	XL	RP	RP2	c.264_271del
30	M	RP	34	RPGR:c.2185G>T	hemizygous	LP	yes	XL	RP	RPGR	c.2185G>T
31	M	RP	22	EYS:c.6544_6547del	homozygous	P	yes	AR/AD	RP	EYS	c.6544_6547del
32	M	RP	30	ABCA4:c.5882G>A	heterozygous	LP	no	AR/AD	RP	ABCA4	c.5882G>A
33	F	RP	33	CNGB1:c.2544del	homozygous	P	yes	AR	RP	CNGB1	c.2544del
34	F	RP	45	RPE65:c.271C>T	homozygous	P	no	AR/AD	RP	RPE65	c.271C>T
35	M	RP	28	CYP4V2:c.1198C>T	homozygous	P	no	AR	Bietti Crystalline Corneoretinal Dystrophy	CYP4V2	c.1198C>T
36	F	RP	30	CRB1:c.2771G>A	homozygous	P	no	AR/AD	RP	CRB1	c.2771G>A
37	F	RP	72	PDE6A:c.2027+1G>C	heterozygous	LP	yes	AR	RP	PDE6A	c.2027+1G>C
38	M	RP	50	CDHR1:c.616delC	homozygous	P	yes	AR	RP	CDHR1	c.616delC
39	M	RP	31	CRB1:c.2771G>A	homozygous	P	no	AR/AD	RP	CRB1	c.2771G>A
40	M	RP	13	RDH12:c.599A>G	homozygous	LP	no	AR/AD	RP	RDH12	c.599A>G
41	F	RP	37	PDE6A:c.304C>A	homozygous	P	no	AR	RP	PDE6A	c.304C>A
42	M	RP	2	TYR:c.1217C>T	homozygous	LP	no	AR	Oculocutaneous Albinism	TYR	c.1217C>T
43	M	RP	45	PDE6B:c.2116A>T	homozygous	P	no	AR/AD	RP	PDE6B	c.2116A>T
44	M	RP	25	CDHR1:c.616del	homozygous	P	no	AR	RP	CDHR1	c.616del
45	F	RP	34	CRX:c.268C>T	homozygous	LP	no	AR/AD	RP	CRX	c.268C>T
46	F	RP	32	CDHR1:c.616del	homozygous	P	no	AR	RP	CDHR1	c.616del
47	F	RP	27	KCNV2:c.339C>A	homozygous	P	no	AR	RP	KCNV2	c.339C>A
48	F	RP	30	ABCA4:c.6316C>T	heterozygous	P	no	AR/AD	RP	ABCA4	c.6316C>T
49	F	RP	54	ABCA4:c.4918C>T	homozygous	P	no	AR/AD	RP	ABCA4	c.4918C>T
50	F	RP	15	EYS:c.2137+1G>A	homozygous	P	no	AR	RP	EYS	c.2137+1G>A
51	M	RP	39	BBS2:c.1864C>T	homozygous	P	no	AR	Bardet-Biedl Syndrome/RP	BBS2	c.1864C>T
52	F	RP	48	CYP4V2:c.332T>C	homozygous	P	no	AR	Bietti Crystalline Corneoretinal Dystrophy	CYP4V2	c.332T>C
53	M	RP	24	USH2A:c.7174_7181del	homozygous	LP	yes	AR	Usher Syndrome	USH2A	c.7174_7181del
54	M	RP	28	RPE65:c.499G>T	homozygous	LP	no	AR/AD	RP	RPE65	c.499G>T
55	M	RP	58	MFRP:c.271C>T	homozygous	P	no	AR	RP	MFRP	c.271C>T
56	M	RP	46	TULP1:c.845del	homozygous	P	yes	AR	RP	TULP1	c.845del
57	F	RP	31	ABCA4:c.5882G>A	heterozygous	LP	no	AR/AD	RP	ABCA4	c.5882G>A
58	F	RP	21	TYR:c.1217C>T	homozygous	LP	no	AR	Oculocutaneous Albinism	TYR	c.1217C>T
59	F	RP	20	BBS5:c.387-2A>G	homozygous	LP	yes	AR	Bardet-Biedl Syndrome/RP	BBS5	c.387-2A>G
60	F	RP	25	USH2A:c.2610C>A	homozygous	P	no	AR	Usher Syndrome	USH2A	c.2610C>A
61	M	RP	60	USH2A, c.5546_5547del, c.14219C>A	compound heterozygous	LP	no	AR	Usher Syndrome	USH2A	c.5546_5547del, c.14219C>A
62	M	RP	10	ABCA4:c.5018+2T>C	homozygous	P	no	AR/AD	RP	ABCA4	c.5018+2T>C
63	F	RP	32	CDHR1:c.338del	homozygous	P	no	AR	RP	CDHR1	c.338del
64	F	Cone-rod dystrophy	20	ABCA4:c.5018+2T>C	heterozygous	P	no	AR/AD	RP	ABCA4	c.5018+2T>C
65	M	RP	48	CRX:c.121C>T	heterozygous	LP	no	AD	RP	CRX	c.121C>T
66	F	RP	23	IMPG2:c.285_286del	homozygous	LP	yes	AR	RP	IMPG2	c.285_286del
67	M	RP	33	ABCA4:c.5018+2T>C	heterozygous	P	no	AR/AD	RP	ABCA4	c.5018+2T>C
68	M	RP	31	CRB1:c.3933+1G>A	homozygous	P	no	AR	RP	CRB1	c.3933+1G>A
69	M	RP	50	RPGR:c.2270_2271del	hemizygous	LP	no	XL	RP	RPGR	c.2270_2271del
70	M	RP	28	ABCA4:c.5018+2T>C	heterozygous	P	no	AR/AD	RP	ABCA4	c.5018+2T>C
71	M	RP	12	ABCA4:c.3113C>T	heterozygous	LP	no	AR/AD	RP	ABCA4	c.3113C>T
72	M	RP	40	CDHR1:c.1448A>G	homozygous	LP	no	AR	RP	CDHR1	c.1448A>G
73	M	RP	7	CLN6:c.551C>G	homozygous	LP	no	AR	Neuronal Ceroid Lipofuscinosis	CLN6	c.551C>G
74	F	RP	12	ABCA4:c.5882G>A	heterozygous	LP	no	AR/AD	RP	ABCA4	c.5882G>A
75	M	RP	5	CDH23:c.5584G>A	homozygous	LP	no	AR	Usher Syndrome	CDH23	c.5584G>A
76	M	RP	15	FAM161A:c.1464G>A	homozygous	P	no	AR	RP	FAM161A	c.1464G>A
77	M	RP	22	ABCA4:c.3287C>T	homozygous	P	no	AR/AD	RP	ABCA4	c.3287C>T
78	F	RP	46	ABCA4:c.5018+2T>C	heterozygous	P	no	AR/AD	RP	ABCA4	c.5018+2T>C
79	M	RP	26	ABCA4:c.5910_5912dupCCT	heterozygous	LP	yes	AR/AD	RP	ABCA4	c.5910_5912dupCCT
80	M	RP	53	ABCA4, c.2941C>T	heterozygous	LP	yes	AR/AD	RP	ABCA4	c.2941C>T
81	M	RP	47	PDE6B:c.1060-1G>T	homozygous	P	no	AR/AD	RP	PDE6B	c.1060-1G>T
82	F	RP	33	RDH12:c.379G>T	homozygous	P	no	AR	RP	RDH12	c.379G>T
83	M	RP	44	EYS:c.6714delT	homozygous	P	no	AR	RP	EYS	c.6714delT
84	M	RP	26	EYS:c.6324C>A	homozygous	P	yes	AR	RP	EYS	c.6324C>A
85	M	RP	9	CRB1, c.4060G>A	heterozygous	LP	no	AR/AD	RP	CRB1	c.4060G>A
86	F	RP	29	TULP1:c.854delC	homozygous	LP	yes	AR	RP	TULP1	c.854delC
87	M	RP	31	PDE6A:c.1264-5_1268del	homozygous	LP	yes	AR	RP	PDE6A	c.1264-5_1268del
88	F	RP	43	RP1:c.5278_5287delAATCCTGGCA	homozygous	LP	no	AR/AD	RP	RP1	c.5278_5287delAATCCTGGCA
89	F	RP	47	EYS:c.6714delT	homozygous	P	no	AR	RP	EYS	c.6714delT
90	M	RP	17	BBS4:c.844A>T	homozygous	LP	yes	AR	Bardet-Biedl Syndrome/RP	BBS4	c.844A>T
91	M	RP	32	LRAT:c.525T>A	homozygous	LP	no	AR	RP	LRAT	c.525T>A
92	F	RP	50	CERKL:c.612_613del	homozygous	LP	yes	AR	RP	CERKL	c.612_613del
93	M	RP	43	CDHR1:c.1381C>T	homozygous	P	no	AR	RP	CDHR1	c.1381C>T
94	F	RP	36	TULP1:c.845delC	homozygous	P	yes	AR	RP	TULP1	c.845delC
95	F	RP	59	MERTK:c.482+1G>T	homozygous	LP	yes	AR	RP	MERTK	c.482+1G>T
96	F	RP	49	CLRN1:c.189C>A	homozygous	P	no	AR	RP	CLRN1	c.189C>A
97	M	RP	41	RDH12:c.464C>T	homozygous	LP	no	AR	RP	RDH12	c.464C>T
98	M	RP	34	RLBP1:c.286_297del	homozygous	P	no	AR	RP	RLBP1	c.286_297del
99	F	RP	23	RDH12:c.464C>T	homozygous	LP	no	AR/AD	RP	RDH12	c.464C>T
100	M	RP	37	ABCA4:c.4139C>T	homozygous	P	no	AR/AD	RP	ABCA4	c.4139C>T
101	M	RP	40	RLBP1:c.398del	homozygous	LP	no	AR	RP	RLBP1	c.398del
102	F	RP	17	ABCA4:c.5714+5G>A	heterozygous	P	no	AR/AD	RP	ABCA4	c.5714+5G>A
103	F	RP	38	PDE6B:c.1670A>G	heterozygous	P	no	AR/AD	RP	PDE6B	c.1670A>G
104	M	RP	9	RDH12:c.379G>T	homozygous	P	no	AR	RP	RDH12	c.379G>T
105	M	RP	28	TULP1:c.1519G>A	homozygous	LP	yes	AR	RP	TULP1	c.1519G>A
106	F	RP	32	ABCA4:c.1622T>C	homozygous	P	no	AR/AD	RP	ABCA4	c.1622T>C
107	M	RP	38	TULP1:c.1574T>G	homozygous	LP	yes	AR	RP	TULP1	c.1574T>G
108	M	RP	46	PDE6A:c.1166C>T	homozygous	LP	no	AR	RP	PDE6A	c.1166C>T
109	M	RP	8	CRB1:c.3806+2T>C	homozygous	LP	yes	AR	RP	CRB1	c.3806+2T>C
110	M	RP	42	BBS2:c.334T>C	homozygous	LP	no	AR	Bardet-Biedl Syndrome/RP	BBS2	c.334T>C
111	M	RP	46	PDE6B:c.1043T>G	homozygous	LP	yes	AR/AD	RP	PDE6B	c.1043T>G
112	M	RP	29	CRB1:c.3469T>A	homozygous	LP	no	AR/AD	RP	CRB1	c.3469T>A
113	M	RP	9	EYS:c.1442G>A	homozygous	P	yes	AR	RP	EYS	c.1442G>A
114	M	RP	31	TULP1:c.349G>A	homozygous	P	no	AR	RP	TULP1	c.349G>A
115	F	RP	48	RDH12:c.379G>T	homozygous	P	no	AR/AD	RP	RDH12	c.379G>T
116	F	RP	32	RHO:c.1033G>A	heterozygous	LP	no	AR/AD	RP	RHO	c.1033G>A
117	F	RP	30	CYP4V2:c.283G>A	homozygous	P	no	AR	Bietti Crystalline Corneoretinal Dystrophy	CYP4V2	c.283G>A
118	F	RP	54	USH2A:c.12067-2A>G	homozygous	P	no	AR	Usher Syndrome	USH2A	c.12067-2A>G
119	M	RP	32	MYO7A:c.6487G>A	homozygous	P	no	AR	Usher Syndrome	MYO7A	c.6487G>A
120	M	RP	26	RDH12:c.444C>A	homozygous	LP	no	AR/AD	RP	RDH12	c.444C>A
121	M	RP	43	PDE6A:c.995delT	homozygous	LP	yes	AR	RP	PDE6A	c.995delT
122	M	RP	43	ABCA4:c.6077T>C	homozygous	P	no	AR	RP	ABCA4	c.6077T>C
123	F	RP	27	MERTK:c.2350-2A>C	homozygous	LP	yes	AR	RP	MERTK	c.2350-2A>C
124	F	RP	29	RPGR:c.2997_2998delGG	homozygous	LP	no	XL	RP	RPGR	c.2997_2998delGG
125	M	RP	29	CYP4V2:c.332T>C	homozygous	P	no	AR	Bietti Crystalline Corneoretinal Dystrophy	CYP4V2	c.332T>C
126	F	RP	15	CERKL:c.1616+1G>T	homozygous	LP	no	AR	RP	CERKL	c.1616+1G>T
127	F	RP	43	ABCA4:c.160T>G	heterozygous	P	no	AR/AD	RP	ABCA4	c.160T>G
128	M	RP	42	CDHR1:c.1516G>T	homozygous	P	no	AR	RP	CDHR1	c.1516G>T
129	M	RP	18	USH2A:c.6127_6128dup	homozygous	P	yes	AR	Usher Syndrome	USH2A	c.6127_6128dup
130	F	RP	42	IMPG2:c.1491delC	homozygous	LP	yes	AR/AD	RP	IMPG2	c.1491delC
131	M	RP	38	BBS1:c.479G>A	homozygous	P	no	AR	Bardet-Biedl Syndrome/RP	BBS1	c.479G>A
132	M	RP	14	USH2A:c.11864G>A, c.11156G>A	compound heterozygous	P	no	AR	Usher Syndrome	USH2A	c.11864G>A, c.11156G>A
133	M	RP	21	TULP1:c.1018A>G	homozygous	LP	yes	AR	RP	TULP1	c.1018A>G
134	M	RP	45	RPGR:c.561delC	hemizygous	LP	yes	XL	RP	RPGR	c.561delC
135	F	RP	43	BEST1:c.107_118delAGTACGAGAACC	heterozygous	LP	no	AR/AD	RP	BEST1	c.107_118delAGTACGAGAACC
136	M	RP	14	CNGB3:c.806T>C	homozygous	LP	no	AR	Achromatopsia	CNGB3	c.806T>C
137	F	RP	28	CERKL:c.612_613del	homozygous	LP	yes	AR	RP	CERKL	c.612_613del
138	F	RP	3	AIRE:c.232T>C	homozygous	P	no	AR/AD	Autoimmune polyendocrinopathy syndrome	AIRE	c.232T>C
139	M	RP	18	CLRN1:c.189C>A	homozygous	P	no	AR	RP	CLRN1	c.189C>A
140	M	RP	4	ABCA4:c.4739T>C	heterozygous	LP	no	AR/AD	RP	ABCA4	c.4739T>C
141	M	RP	27	HMCN1:c.254C>G	heterozygous	LP	yes	AD	Age-Related Macular Degeneration	HMCN1	c.254C>G
142	F	RP	14	ABCA4:c.5882G>A	heterozygous	LP	no	AR/AD	RP	ABCA4	c.5882G>A
143	M	RP	28	CNGA3:c.1588G>A	heterozygous	LP	yes	AR	Achromatopsia	CNGA3	c.1588G>A
144	M	RP	4	COL4A1:c.3592G>C	heterozygous	P	no	AD	Retinal Arterial Tortuosity	COL4A1	c.3592G>C
145	M	RP	59	CTNNB1:c.101G>A	heterozygous	LP	no	AD	Exudative Vitreoretinopathy	CTNNB1	c.101G>A
146	M	RP	60	CTNNB1:c.110C>T	heterozygous	LP	no	AD	Exudative Vitreoretinopathy	CTNNB1	c.110C>T
147	F	RP	67	CTNNB1:c.110C>T	heterozygous	LP	no	AD	Exudative Vitreoretinopathy	CTNNB1	c.110C>T
148	M	RP	28	RDH12:c.464C>T	heterozygous	LP	no	AR/AD	RP	RDH12	c.464C>T
149	M	RP	28	RPGR:c.2404_2406delCCA	hemizygous	LP	no	XL	RP	RPGR	c.2404_2406delCCA
150	F	RP	32	BBS10:c.930dupA	homozygous	LP	yes	AR	Bardet-Biedl Syndrome/RP	BBS10	c.930dupA
151	M	RP	17	ABCA4:c.4139C>T	heterozygous	P	no	AR/AD	RP	ABCA4	c.4139C>T
152	M	RP	24	COL2A1:c.1690G>A	heterozygous	LP	no	AD	RP	COL2A1	c.1690G>A
153	M	RP	11	RP1L1, c.7076C>G	heterozygous	LP	yes	AR/AD	RP	RP1L1	c.7076C>G
154	M	Neuromuscular Disease	16	ABCA4:c.5882G>A	homozygous	LP	no	AR/AD	RP	ABCA4	c.5882G>A
155	M	RP	17	PCDH15:c.4789_4792dup	homozygous	LP	yes	AR	Usher Syndrome	PCDH15	c.4789_4792dup
156	M	RP	16	COL2A1:c.1052G>T	heterozygous	LP	no	AD	RP	COL2A1	c.1052G>T
157	F	BBS	8	BEST1:c.470dupT	heterozygous	LP	yes	AR/AD	RP	BEST1	c.470dupT
158	F	Neuromuscular Disease	33	SLC45A2:c.1115G>A	homozygous	LP	yes	AR	Oculocutaneous Albinism	SLC45A2	c.1115G>A
159	M	Neuromuscular Disease	63	ABCA4:c.5882G>A	heterozygous	LP	no	AR/AD	RP	ABCA4	c.5882G>A
160	M	RP	20	ADAM9:c.647T>C	homozygous	LP	yes	AR	RP	ADAM9	c.647T>C
161	M	Aniridia	21	PAX6:c.241C>T	heterozygous	LP	no	AD	Coloboma	PAX6	c.241C>T
162	F	RP	23	LRP5:c.1096G>A	heterozygous	LP	no	AR/AD	RP	LRP5	c.1096G>A
163	M	Aniridia	10	RP1L1:c.5959C>T	heterozygous	LP	no	AR/AD	RP	RP1L1	c.5959C>T
164	M	RP	33	ABCA4:c.5882G>A	heterozygous	LP	no	AR/AD	RP	ABCA4	c.5882G>A
165	M	RP	41	SLC6A6:c.228_229del	homozygous	LP	yes	AR/AD	RP	SLC6A6	c.228_229del
166	F	Neuromuscular Disease	25	ABCC6:c.2294G>A	heterozygous	LP	no	AR/AD	RP	ABCC6	c.2294G>A
167	M	RP	31	PDE6A:c.2507-1G>T	homozygous	LP	yes	AR	RP	PDE6A	c.2507-1G>T
168	F	Neuromuscular Disease	5	EYS:c.2137+1G>A	heterozygous	P	no	AR	RP	EYS	c.2137+1G>A
169	F	RP	34	PDE6A:c.2507-1G>T	heterozygous	LP	yes	AR	RP	PDE6A	c.2507-1G>T
170	F	RP	41	CYP4V2:c.332T>C	homozygous	P	no	AR	Bietti Crystalline Corneoretinal Dystrophy	CYP4V2	c.332T>C
171	F	RP	16	POMT1:c.1939G>A	homozygous	P	no	AR	RP	POMT1	c.1939G>A
172	M	RP	49	INPP5E:c.1073C>T	homozygous	LP	no	AR	MORM Syndrome	INPP5E	c.1073C>T
173	F	RP	11	ABCA4:c.5882G>A	homozygous	LP	no	AR/AD	RP	ABCA4	c.5882G>A
174	M	RP	31	CHM:c.808C>T	hemizygous	P	no	XL	Choroideremia	CHM	c.808C>T
175	M	RP	38	SLC45A2:c.386-1G>A	homozygous	P	yes	AR	Oculocutaneous Albinism	SLC45A2	c.386-1G>A
176	M	RP	25	AHI1:c.3196C>T	homozygous	LP	no	AR	RP	AHI1	c.3196C>T
177	M	RP	4	SLC24A5:c.216T>G	homozygous	P	no	AR	Oculocutaneous Albinism	SLC24A5	c.216T>G
178	F	RP	31	SLC45A2:c.386-1G>A	homozygous	P	yes	AR	Oculocutaneous Albinism	SLC45A2	c.386-1G>A
179	M	RP	18	SLC24A5:c.216T>G	homozygous	P	no	AR	Oculocutaneous Albinism	SLC24A5	c.216T>G
180	M	Usher	1	CC2D2A:c.3584del	homozygous	P	no	AR	RP	CC2D2A	c.3584del
181	M	Neuromuscular Disease	43	SPG7:c.233T>A	homozygous	P	no	AR	Spastic paraplegia	SPG7	c.233T>A
182	M	Neuromuscular Disease	32	PDE6B:c.1670A>G	heterozygous	P	no	AR/AD	RP	PDE6B	c.1670A>G
183	F	RP	34	ABCA4:c.5882G>A	heterozygous	LP	no	AR/AD	RP	ABCA4	c.5882G>A
184	F	OCA	70	TYR:c.1217C>T	homozygous	LP	no	AR	Oculocutaneous Albinism	TYR	c.1217C>T
185	M	RP	16	RDH5:c.712G>T	heterozygous	LP	no	AR/AD	RP	RDH5	c.712G>T
186	F	RP	32	EYS:c.4045C>T	homozygous	P	no	AR	RP	EYS	c.4045C>T
187	M	RP	57	SCAPER:c.987+1G>A	homozygous	LP	yes	AR	RP	SCAPER	c.987+1G>A
188	M	BBS	28	WFS1:c.1673G>A	heterozygous	LP	no	AR/AD	Wolfram Syndrome	WFS1	c.1673G>A
189	F	Neuromuscular Disease	24	AHI1:c.3032C>G	homozygous	P	no	AR	RP	AHI1	c.3032C>G
190	M	RP	43	LRAT:c.525T>A	homozygous	LP	no	AR	RP	LRAT	c.525T>A
191	M	Optic Atrophy	11	TMEM126A:c.292_293del	homozygous	LP	yes	AR	RP	TMEM126A	c.292_293del
192	M	RP	22	ABCA4:c.5882G>A	heterozygous	LP	no	AR/AD	RP	ABCA4	c.5882G>A
193	F	Glaucoma	72	MYOC:c.1130C>T	heterozygous	P	no	AD	Glaucoma	MYOC	c.1130C>T
194	M	RP	43	USH2A:c.6127_6128dup	homozygous	P	yes	AR	Usher Syndrome	USH2A	c.6127_6128dup
195	F	Optic Atrophy	8	RPGRIP1:c.1447C>T	homozygous	P	no	AR	RP	RPGRIP1	c.1447C>T
196	M	Optic Atrophy	9	CNGA3:c.1252C>T	homozygous	P	no	AR	RP	CNGA3	c.1252C>T
197	F	Neuromuscular Disease	50	PAX6:c.1138del	heterozygous	LP	no	AD	Coloboma	PAX6	c.1138del
198	M	LCA	14	CEP290:c.2218-2A>T	homozygous	LP	no	AR	RP	CEP290	c.2218-2A>T
199	F	Usher	32	USH2A:c.2610C>A	homozygous	P	no	AR	Usher Syndrome	USH2A	c.2610C>A
200	F	RP	16	TULP1:c.409G>T	homozygous	LP	no	AR	RP	TULP1	c.409G>T
201	M	BBS	60	AIRE:c.769C>T	heterozygous	LP	no	AR/AD	Autoimmune polyendocrinopathy syndrome	AIRE	c.769C>T
202	M	BBS	1	DYNC2H1:c.5960C>T	homozygous	LP	yes	AR	RP	DYNC2H1	c.5960C>T
203	F	RP	3	TYR:c.1217C>T	homozygous	LP	no	AR/AD	Oculocutaneous Albinism	TYR	c.1217C>T
204	M	BBS	13	BBS2:c.256_278dup	homozygous	P	no	AR	Bardet-Biedl Syndrome/RP	BBS2	c.256_278dup
205	F	RP	28	ABCA4:c.2588G>C	heterozygous	LP	no	AR/AD	RP	ABCA4	c.2588G>C
206	M	BBS	23	ZNF408:c.188_194delTTGGCCC	heterozygous	LP	yes	AR/AD	RP	ZNF408	c.188_194delTTGGCCC
207	F	RP	11	CYP4V2:c.253C>T	homozygous	LP	no	AR	Bietti Crystalline Corneoretinal Dystrophy	CYP4V2	c.253C>T
208	M	RP	12	AP3B2:c.1283-2A>G	homozygous	LP	yes	AR/AD	RP	AP3B2	c.1283-2A>G
209	F	Usher	4	USH2A:c.7524del	homozygous	P	no	AR	Usher Syndrome	USH2A	c.7524del
210	F	RP	19	PEX1:c.1099delC	homozygous	P	no	AR	Peroxisome Biogenesis Disorder	PEX1	c.1099delC
211	F	RP	14	PEX1:c.1099delC	homozygous	P	no	AR	Peroxisome Biogenesis Disorder	PEX1	c.1099delC
212	F	RP	19	RP1L1:c.7076C>G	heterozygous	LP	yes	AR/AD	RP	RP1L1	c.7076C>G
213	F	RP	12	TREX1:c.838_855delCCACTGGGTCTGCTGGCC	homozygous	P	no	AR	RP	TREX1	c.838_855delCCACTGGGTCTGCTGGCC
214	M	RP	4	CEP290:c.5932C>T	homozygous	P	no	AR	RP	CEP290	c.5932C>T
215	M	RP	14	INPP5E:c.1301G>A	homozygous	P	no	AR	RP	INPP5E	c.1301G>A
216	M	RP	8	COL2A1:c.4135C>T	heterozygous	LP	no	AR/AD	RP	COL2A1	c.4135C>T
217	F	RP	16	NR2E3:c.932G>A	heterozygous	LP	no	AR/AD	RP	NR2E3	c.932G>A
218	M	RP	28	SNRNP200:c.2951T>A	heterozygous	LP	yes	AR/AD	RP	SNRNP200	c.2951T>A
219	M	RP	4	CTNNB1:c.1981C>T	heterozygous	P	no	AR/AD	RP	CTNNB1	c.1981C>T
220	F	RP	4	MYO7A:c.4025G>A	homozygous	LP	no	AR	Usher Syndrome	MYO7A	c.4025G>A
221	F	RP	28	MYO7A:c.6487G>A	homozygous	P	no	AR	Usher Syndrome	MYO7A	c.6487G>A
222	M	RP	28	CDH23:c.5584G>A	homozygous	P	no	AR	Usher Syndrome	CDH23	c.5584G>A
223	F	Neuromuscular Disease	2	PPT1:c.566C>G	homozygous	LP	no	AR	Neuronal Ceroid Lipofuscinosis	PPT1	c.566C>G

*ABCA4* emerged as the gene with the highest frequency of mutations. A total of 38 mutations were identified in the *ABCA4* gene, accounting for about 17% of the observed cases. The investigation identified the presence of the recurrent *ABCA4*:c.5882G>A variant in 11 individuals, accounting for 5% of the total sample. Additionally, the *ABCA4*:c.5018+2T>C variant was revealed in five unrelated patients, representing 2.2% of the study population (Figure 1). The identified variants with pathogenic and likely pathogenic effects were confirmed using Sanger sequencing in both the patient and all other accessible family members. A total of 58 novel mutations were identified, all of which were previously unreported in existing literature. These genetic sequences are classified as evolutionarily highly conserved areas, and computational methods have demonstrated their association with disease. It is postulated that nonsense and frameshift mutations have the potential to induce nonsense-mediated mRNA decay.

## Discussion

This paper presents comprehensive clinical and molecular information about a cohort of 223 people with mutations in genes associated with IRD. The considerable genetic variation exhibited by this illness poses a substantial challenge in the context of molecular diagnostics within clinical settings. Conventional approaches to individual gene screening pose challenges and are unlikely to provide a comprehensive understanding of the complete range of mutations within the patient group. NGS is a high-throughput methodology that exhibits the capability to rapidly sequence extensive collections of genes, hence generating substantial volumes of data. The utilization of this technology has emerged as a potent method for comprehensively understanding mutation profiles in illnesses characterized by heterogeneity.

Inheritance of IRD occurs through three modes: XL, autosomal recessive (AR), and autosomal dominant (AD). The incidence of the XL variant, which is more commonly expressed in men, leads to a slightly higher prevalence among males than females [3]. In our study, the number of male participants (n = 130, 58.3%) exceeded that of female participants (n = 93, 41.7%).

The top five genes identified by RetinoGenetics are *ABCA4, USH2A, RPGR, WFS1, *and* CRB1. ABCA4* was identified as the predominant variant in our study. Nevertheless, the gene that was observed with the second highest frequency was *EYS*. According to RetNet and existing literature, there have been numerous observations of EYS mutations in France, Spain, China, and Pakistan [12]. The most prominent genes in our study include *ABCA4* (17%), *EYS* (5%), *USH2A* (4%), *TULP1* (4%), *RDH12* (4%), *CDHR1* (4%), *PDE6A* (3%), *CRB1* (3%), *CYP4V2* (3%), *RPGR* (2%), and *RPE65* (2%). The prevalence of *CRB1* mutations is likewise shown to be high in the population of Spain. According to RetNet, the prevalence of *TULP1, RDH12, CDHR1, *and* CYP4V2 *mutations are either unknown or below 1%. However, it has been frequently observed in our patients with IRD [2].

Extensive research is presently being conducted on gene therapy as a potential treatment option for a range of retinal degenerative disorders. Published clinical trials have demonstrated varying levels of efficacy in individuals affected by *RPE65*-related LCA, severe early-childhood-onset retinal dystrophy (SECORD), and disorders characterized by infantile-onset or early-childhood onset closely linked to RP [13-15].

The clinical presentations observed in individuals with IRD exhibit a wide range of severity, and there is no discernible correlation between the specific gene or mutation responsible for the condition and the extent of symptoms. The clinical underdiagnosis of some kinds of IRD can be attributed to the diverse range of phenotypes associated with this condition [16]. Various mutations within a single gene can result in different diseases, as exemplified by the case of *ABCA4*. This gene is implicated in several disorders, including autosomal-recessive Stargardt disease 1; RP 19; Retinal dystrophy, early-onset severe; Fundus flavimaculatus; Cone-rod dystrophy 3; and autosomal-dominant Macular degeneration, age-related, 2 (MIM 601691). Various genetic mutations, including those in *ABCA4, RPGR, PDE6B, *and* RHO*, have been identified as causative factors for RP and other retinal illnesses.

Three areas of uncertainty exist about the concept of IRD. One of the primary factors to consider is phenotypic variability. Patients with the same mutations had a spectrum of phenotypic symptoms, demonstrating varied degrees of severity ranging from mild to severe [6]. A subset of people within our sample population had a more prominent phenotype in comparison to their parents or siblings, although carrying an identical mutation. The prevalence of *ABCA4* and *PDE6B* mutations has been predominantly reported in patients. The wide range of clinical variations found in people can be attributed to several factors, such as variable expressivity, incomplete penetrance, and the impact of mutant alleles on the functionality of wild-type proteins. There is a potential for additional genes' involvement in the diverse range of phenotypes, serving as modifiers. The presence of significant variation within families suggests that genetic modifiers, as well as epigenetic or environmental influences, might potentially play a role in the findings that were presented. Hence, it is imperative that thorough sequencing analysis utilizing NGS be considered as the principal approach for gene screening in IRD.

One further concern is the challenge of differentiating between AD and AR disorders. Certain genetic alterations demonstrate a pattern of inheritance characterized by dominance and recessiveness, which adds complexity to our comprehension of the association between genotype and phenotype, as well as the mode of inheritance [6]. This observation was also apparent in our study participants, namely in those individuals who had mutations in the *ABCA4* and *PDE6B* genes. The clinical underdiagnosis of some kinds of IRD can be attributed to the wide range of phenotypic variations observed within this group [16]. Determining the mode of inheritance holds significant importance in the field of genetic counseling, as well as in the identification of additional family members who may be at risk. Additionally, it plays a crucial role in prenatal and preimplantation genetic diagnostics. An accurate diagnosis of IRD will result in the provision of genetic counseling services for families, as well as the implementation of more efficient treatment approaches.

Another of the primary challenges is accurately predicting the phenotype and prognosis by considering mutations and zygosity. It is anticipated that individuals with non-functional early truncating mutations will exhibit severe clinical symptoms. Based on this assumption, it would be expected that the majority of our patients will exhibit a severe phenotype. However, it is seen that these individuals display varying phenotypes, even within the same family. The precise nature of the connection between genetic defects, the causes of diseases, and the observable clinical manifestations is now not fully understood.

## Conclusions

The findings of this study unveiled several previously unidentified mutations in IRD and provided evidence of substantial diversity in the relationship between genotypes and phenotypes in the illness. Significantly, the findings given in this study provide empirical proof that the use of comprehensive NGS approaches in clinical settings might contribute to a deeper comprehension of the intricate manifestations of IRD. A total of 58 novel variations have been added to the expanding collection of mutations linked with IRD. The discovery of several novel disease-causing variants across several populations enhances our comprehension of the associations between genotypes and phenotypes, paving the way for novel treatment approaches. Additional extensive functional investigations are required, utilizing larger cohorts, to explore the impact of mutations on IRD in terms of inheritance patterns and phenotypic heterogeneity.
